# Estimation of effective half life of clearance of radioactive Iodine (^131^I) in patients treated for hyperthyroidism and carcinoma thyroid

**DOI:** 10.4103/0972-3919.72686

**Published:** 2010

**Authors:** R Ravichandran, JP Binukumar, Amal Al Saadi

**Affiliations:** Medical Physics Unit, National Oncology Center, Royal Hospital, Muscat-111, Oman; 1 Department of Nuclear Medicine, National Oncology Center, Royal Hospital, Muscat-111, Oman

**Keywords:** Carcinoma thyroid, body burden, effective half life, iodine-131

## Abstract

**Background:**

In medical applications of radioisotopes, for calculations of whole body doses and radiation safety applications, there is a need to estimate radioactive body burden. Local recommendations in Oman stipulate the need for hospitalization of patients treated for radioactive-iodine (^131^ I) with activities above 400 MBq.

**Materials & Methods:**

A study of body burden estimation from sequentially measured exposure rates from patients treated for carcinoma thyroid and hyperthyroidism was undertaken. A digital auto-ranging beta gamma survey instrument calibrated for measurement of exposure rates is used in this study.

**Results:**

The mean measured exposure rates at 1 m in *μ*Sv/h immediately after administration and at 24 h intervals are used for estimation of effective half time of clearance of administered activity. For patients with post-operative thyroid carcinoma, the variation of body burden with time post-administration indicated tri-exponential clearance pattern, with T_½eff_ values 14.4 h, 22 h, and 41.3 h. For patients treated for thyrotoxicosis, the body burden showed slow delayed clearance with a T_½eff_ – 111.4 h, and exposure rates did not show appreciable fall off after 48 h.

## INTRODUCTION

Treatments for ablation of functioning thyroid for management of hyperthyroidism and for the elimination of post-surgical residual thyroid tissues in differentiated carcinoma of thyroid by administration of radioactive iodine (RA^131^I) are well-established procedures in clinical nuclear medicine. The administered radioactive iodine is absorbed in the functioning thyroid tissue and gets eliminated mainly from the bowel, resulting in whole body dose in addition to being taken up in the glandular tissues of thyroid. The effective half life for clearance of radioactive body burden for normal human subjects is about 5.5 days (about 132 h) which is useful in health physics applications, to calculate the radiation dose received by the thyroid and total body, from the beta and gamma emissions originating from the radioactive iodine. In case of patients treated for carcinoma thyroid, because of surgical removal of thyroid tissues, elimination of administered I-131 radioactivity clears at a faster rate, thereby allowing treatments with large amounts of I-131 as high as 1.85 GBq to 5.5 GBq (50-150 mCi).

The assessment of external radiation exposures from patients at known geometry using radiation monitors is one of the accepted standard methods for estimation of radioactive body burden.[1–5] Present regulations in Oman[6] indicates the need for hospitalization of patients above 400 MBq administered activities of RA^131^I. We have only two isolation rooms connected with delay tank facility for I-131 treatments. This led to a waiting list for treatments of thyroid cancers in the oncology center. A recent report from Oman[7] has indicated that with good radiation safety instructions to patients and relatives, thyrotoxic patients may be treated as outpatients, provided the patient is physically and mentally fit enough to comply with given instructions. To address the isolation of Omani patients treated with RA^131^I in the wards, justifying out-patient treatments for patients with thyrotoxicois and making necessary recommendations regarding their retention period of Ca. thyroid patients, we have analyzed the records of external exposure rates measured from patients who underwent radio-iodine therapy both for differentiated carcinoma thyroid and thyrotoxicosis.

## MATERIALS AND METHODS

### Patients

The patients who received RA^131^I were referred from endocrinology department of the Royal Hospital based on accepted protocols [Table 1]. Patients were accepted for their treatments after obtaining informed consent after these patients were properly explained by the nuclear medicine consultants about the procedure, and possible associated risks. The patients treated during the period 2006 to 2009 were taken up for the study.

**Table 1:** Standard operating procedure of nuclear medicine department

Procedure for I-131 administration (thyrotoxicosis)		Procedure for I-131 administration (Ca. Thyroid)	
Referral from	Dept. of nuclear. medicine	Referral from	Dept. of nuclear. medicine
Endocrinology	Informed consent instructions (Arabic/ English), confirm patients awareness, inform patients preparation appointment/Order I-131	Endocrinology	Surgical details/Histology near total thyroidectomy 4 weeks elapsed after surgery informed consent instructions (Arabic/English) Confirm patients awareness inform patients preparation. Stop thyroxine 4 weeks before I-131 therapy appointment/Order I-131
Patient preparation	Stop iodine containing drugs or food 2 weeks before I-131 administration. Stop antithyroid drug for 5 days	Date of admission	Confirm patient not pregnant TSH should be >30 I.U Check blood counts On empty stomach >4h Anti-emetic medication
Date of admission	Confirm patient not pregnant. Stop lactation permanently. Check blood counts. On empty stomach	Dose administration	Nuclear medicine physician nuclear medicine physicist instructions/Administation confirm consignment No. preparation of records monitor exposure rates lemon drops to simulate saliva. Encourage fluid uptakes
Dose administration	Nuclear medicine physician nuclear medicine physicist instructions/administation confirm consignment No. preparation of records monitor exposure rates	Dose of RA131I	3.7 GBq on first ablation 5.55GBq for 2nd ablation 7.50GBq for metastases Start higher dose if large tumour or lymphnode +ve
Dose of RA131I	555MBq (15mCi)	Discharge of patients	at < 10μSv/h 1m exp. rate whole body scan + scan of Neck. Patients are discharged by Endocrino- logists on the advice of Radiation Physicist. Give discharge Instructions. Not to become pregnant or fathering for 6 months
Discharge of patients	at < 10μSv/h 1m exp. rate Give discharge instructions	Room monitoring	Measure radioactive levels waste disposal, Records
Room monitoring	Measure radioactive levels Bed, linen, bathroom, sink, Floor, TV monitot, door etc. Waste disposal, Records		

### Administration of RA^131^I

The radioactive iodine is in the form of capsules supplied by GE Healthcare, Amersham, UK. The administrations were carried out on Mondays, and the activities were ordered with reduced activities with reference dates on Thursdays. The radioactive strengths (activity) of the capsules were assayed in Mark V, CalRad Isotope Calibrator (Model 34-164, Nuclear Associates, USA) immediately after arrival of consignment. The containers are labeled with suitable identifications, and activity certificates are prepared in the department. The work flow relating to RA^131^I administrations followed in the department is shown in Table 2.

**Table 2:** Work Flow regarding radioiodine administration

Consignment arrival	Receipt, acknowledgment, inspection activity calibration, labelling, storage inventory, activity certification
Prescription	Transportation to ward, temporary storage
Check patient’s details	Check consignment details
Instructions to the patient	Administration of activity
Patient’s Monitoring	Exposure rates, area monitoring
Preparation of Records	Entries of activity details, display of radiation levels Exposure rates at 24h intervals till discharge
Providing instructions	On the day of discharge

### Measurement of exposure rates

Radiation monitoring is carried out using a wide range beta gamma survey meter, auto-ranging with digital reading facility made up of built-in ionization chamber (Inovision, USA). This is hand held type with measuring range 0.1 μSv/h to 100 Sv/h. The exposure rates are measured at 5 cm distance at stomach and neck levels; and at 1 m and 2 m distances with patient sitting on the hospital bed. These measurements are recorded on the first and subsequent days till discharging the patient. Patients are allowed to go home when their exposure rates fall below 10μSv/h (1.0 mR/h) at 1 m distance. This may correspond to retention of residual body burden of about 170 MBq (4.6 mCi).

## RESULTS

**Figure 1:**
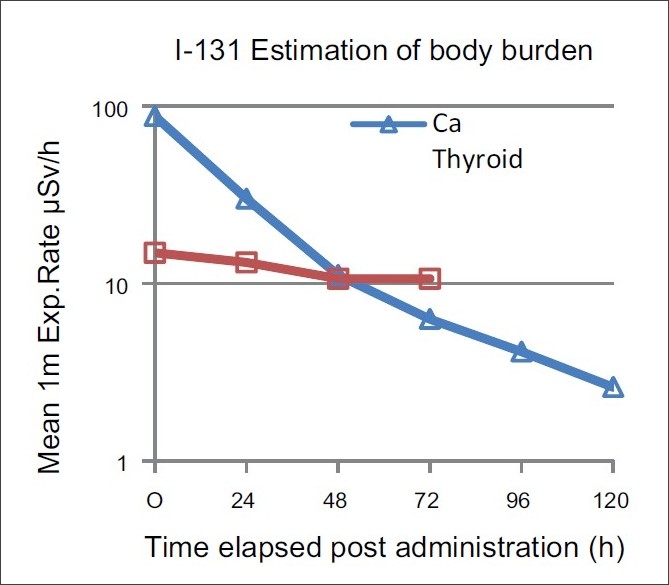
Patterns of Clearance of body burden with administration of RA^131^I

Table 3 shows the number of patients who received treatments for Ca. thyroid and thyrotoxicosis along with the activities of Ra^131^I administered. The mean activities administered were 4.19 GBq and 574.7 MBq for these groups of patients respectively. Table 4 shows the measured mean exposure rates and standard deviation. The number of patients is indicated in brackets. The semi-log plot of these mean exposure rates [Figure 1] gave two distinct patterns of clearance of radioactive body burdens. For patients with post-operative Ca.thyroid, the variation of body burden with time post-administration indicated a tri-exponential pattern. The three slopes of the plots yielded T_½eff_ values 14.4 h, 22.0 h and 41.3 h. For patients treated for thyrotoxicosis, the body burden showed slow rate of clearance with a T_½eff_ =111.4 h, and the exposure rate at 1 m did not show appreciable change after 48 h [Table 4].

**Table 3:** Details of administered activity

Diagnosis	Administered activity GBq/ MBq	Number of patients for years			
		2006	2007	2008	2009
Ca. Thyroid	Range 2.04 to 9.3 GBq 4.363 + 1.17 GBq (n=69)	7	13	25	24
Thyrotoxicosis	Range 479-627 MBq 574.7 + 27.3 MBq (n=50)	12	23	12	3

**Table 4:** Measured Exposure rates in μSv/h at 1m from patients post administration of I-131

Patients	0h	24h	48h	72h	96h	120h
Ca. Thyroid	Range34-184 88.0+37.6 (n=69)	Range10-75 30.1+21.6 (n=69)	Range2.3-37 11.6+7.4 (n=69)	Range1.0-25 6.3+6.4 (n=43)	Range0.3-20 4.12+ 4.6 (n=26)	Range0.3-13 2.6+2.9 (n=29)
Thyrotoxicosis	Range4.5-34 14.9+6.0 (n=49)	Range4.2-27 13.1+5.0 (n=45)	Range2.3-23 10.6+5.0 (n=41)	Range 5.4-17 10.6+4.0 (n=16)	–	–

## DISCUSSION

Our mean exposure rates at 1 m immediately following administration of activity were 88.0 μSv/h (SD 37.6) and 14.9 μSv/h (SD 6), respectively, for the Ca. thyroid and thyrotoxic patients. The results of 1 m exposure rates for thyrotoxic patients 23.4 ±; 6.3 μSv/h as reported by Ibtisaam and Bererhi[7] are of the same magnitude. The mean activity of thyrotoxic patients is 574.7 MBq in the present work, to the mean activity of 609.8 MBq reported by these authors. For the patients with thyrotoxicosis, the mean half time of clearance of body burden in our work is T_½eff_ = 111.4 h which is of the order of magnitude quoted by Meredith and Massey,[8] which is 5.5 days (about 136 h).

**Figure 2:**
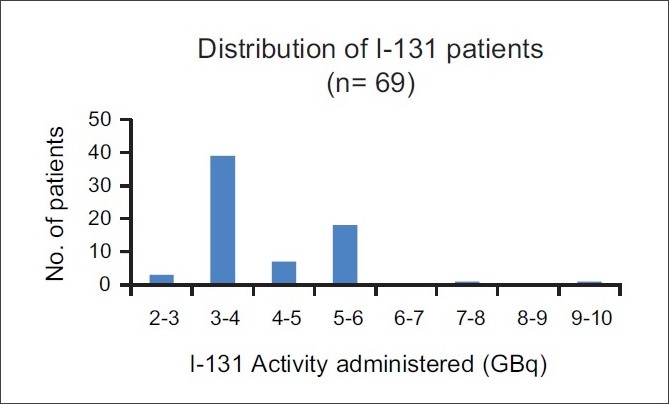
Details of administered activities in Ca Thyroid patients

In our study, the exposure rate at 1 m is averaged over measured values and represented along with the range. This is because of many variables, such as administered activities [Figure 2], various age groups of patients ranging from 18 to 73 years, both sex and different patient weights. For health physics purposes, these values still give an estimate of the risk involved in terms of radiation levels encountered during isolation of these patients.

In the Ca. thyroid patients in the present work, an estimated effective half time of clearance of 14.4 h for body burden is in good agreement with the earlier reported value 11.2-15.4 h[5] in Indian population, and the values reported by Barrington *et al*,[4] viz. 9.4 h, 13.4 h and 12 h. Thomas *et al*,[1] compared three methods of body burden estimation viz. 1) urine assay 2) prediction based on a pre-therapy diagnostic work up method using 74 MBq (2 mCi) 3)external exposure rate measurements during actual treatments. They opined that direct external exposure rate surveys showed the potential for being an accurate, reliable and relatively safe method of monitoring patient’s I-131 body burden. From Table 4, it is clearly seen that the exposure rate around the patient treated for thyrotoxicosis remains unchanged after 48 h, seldom reaching the value of 10 μSv/h (1 mR/h) at 1 m because of retention of activity by toxic thyroid.

The Nuclear Regulatory Commission (NRC) specifies patient’s discharge when the body burden falls below about 1100 MBq (30 mCi). However, based on the social and cultural reasons and living conditions in their home, 1100 MBq I-131 appears to be a higher activity limit in Oman. A lower limit of 400 MBq for the patients to be hospitalized (as per prevailing MOH recommendations)[6] will indicate need for hospitalization of all the patients treated for thyrotoxic disorders (above 370MBq). This will make the need for creation of more isolation rooms, which is not practical. Our body burden measurements in thyrotoxic patients treated in the last four years clearly indicate an effective half time of clearance around 114 h (about five days) and no further clearance after 48 h post administration. In this context, it appears that there is no justification in hospitalizing these thyrotoxic patients post administration of I-131 to reduce their body burden to permissible limits, with the presence of healthy thyroid, concentrating bulk of administered activities because of high uptakes. Hospitalization of a few patients for thyrotoxicosis treatments may therefore be selectively restricted to some cases who have infant children around or many inmates living in small homes, justifying reduction of public exposures to permissible levels.
